# Progressive lateralization and constant hip geometry in children with DDH, NDH, and LCPD following hip reconstructive surgery: a cohort study of 73 patients with a mean follow-up of 4.9 years

**DOI:** 10.1007/s00402-021-04227-x

**Published:** 2021-10-23

**Authors:** Lorenz Pisecky, Gerhard Großbötzl, Manuel Gahleitner, Stella Stevoska, Christian Stadler, Christina Haas, Tobias Gotterbarm, Matthias C. Klotz

**Affiliations:** 1 Department for Orthopaedics and Traumatology, Kepler University Hospital GmbH, Krankenhausstrasse 9, 4020 Linz, Austria; 2 Johannes Kepler University Linz, Altenberger Strasse 69, 4040 Linz, Austria

**Keywords:** DDH, NDH, LCPD, Combined osteotomy, Femur, Ilium, VDRO, Salter, Pemberton, Chiari, Open reduction, Soft-tissue release

## Abstract

**Background:**

Pelvic and femoral osteotomies have been effective methods to treat developmental dysplasia of the hip (DDH), neurogenic dislocation of the hip (NDH), and Legg–Calvé–Perthes disease (LCPD). The aim of this study was to evaluate the mid-term results after hip reconstruction in children with DDH, NDH, and LCPD.

**Methods:**

In a retrospective study, X-rays of 73 children (2–19 years) with DDH, NDH, and LCPD were measured before, 3 months, and at final follow-up (FU) after hip reconstructive surgery (open reduction, and femoral and/or pelvic osteotomy ± soft-tissue procedures between 2008 and 2018). Measurement of hip geometry included acetabular index (AI), center-edge angle (CE), and Reimers migration index (RMI). Mean follow-up time at final FU was 4.9 years. *P* value was set *P* < 0.05.

**Results:**

After surgery (femoral osteotomy: 84 hips, Salter innominate osteotomy: 21 hips, Pemberton osteotomy: 30 hips, open reduction: 28 hips, Chiari osteotomy: 4 hips, and soft-tissue release: 24 hips), hip geometry parameters improved significantly. Nevertheless, at final FU, there was deterioration in hip geometry with femoral head lateralization (RMI) compared to the data at 3 months after surgery (RMI: preop/3 months/final FU: 40.6 ± 16.1%/6.1 ± 9.0/15.4 ± 16.0%; CE: 11.3° ± 20.0°/30.2° ± 9.5°/27.9 ± 15.4°; AI: 28.8° ± 9.6°/19.1° ± 7.6°/18.3 ± 7.6°). Sub-group analysis did not show differences concerning the progression of RMI in DDH, NDH, and LCPD at final FU. Regardless of basic disease, the lateralization was observed in all three groups (DDH, NDH, and LCPD) and statistically significant comparing X-rays 3 months postoperatively to maximum follow-up (DDH; NDH; LCPD: 2.7 ± 6.8%/7.6 ± 10.1%; 13.7 ± 15.3%/22.8 ± 19.8%; 1.7 ± 4.1%/14.9 ± 11.3%). Additional soft-tissue release techniques in patients with DDH or NDH did not show postoperative differences with statistical significance. Concerning surgical techniques, a connection between the lower RMI and the procedure of osteotomy of the ilium was found. In 25 patients, (34%) complications were observed: superficial skin lesions in 8, deep skin lesions in 3, contraction of adductors in 3, subluxation in 2, dislocations of the cast in 2, osteonecrosis of the femoral head in 2 cases, reluxation in 1, infection of the implanted plate in 1, compliance problem in 1, delayed bone healing in 1, and contraction of knee flexors in 1 case.

**Discussion:**

The basic results of this study show a significant improvement of hip geometry at a follow-up of 4.9 years and prove findings of previously published literature. Moreover, the study was able to show a progression of RMI in all patient groups, which have undergone reconstructive surgery, despite basic hip geometry data (AI, CE angle) did not change. Those findings were independent from underlying pathology. Complications were counted in 34% of the patients and involved all known adverse events after hip reconstructive surgery. This makes clear why annual follow-up checks are needed not to miss the right indication for revision surgery.

**Clinical relevance:**

**Evidence level**: Level IV, case series.

**Trial registration:** This manuscript is part of a prospective randomized clinical trial, registered in the German Clinical Trials Register DRKS-ID: DRKS00016861.

## Background

Several developmental and acquired conditions of the infant and juvenile hip joint, such as developmental dysplasia of the hip (DDH), neurogenic dislocation of the hip (NDH), as well as Legg–Calvé–Perthes disease (LCPD), require surgical therapy to avoid persistent impairment in walking, standing, and sitting [1].

In addition to DDH, young patients with NDH and LCPD benefit from surgical reconstruction of the hip when improvement of the containment is necessarily needed to prevent further dislocation (NDH) or to support the rebuilding of the femoral head (LCPD) [2–4].

The reconstruction of the pelvic geometry under usage of femoral and pelvic osteotomies is widely accepted to be a proper treatment for DDH, NDH, and LCPD, despite the techniques and algorithms of stepwise procedures are matters of ongoing discussions.

The majority of patients is in need for femoral as well as pelvic osteotomies to acquire sufficient congruency of the hip. Available literature shows a broad variety of osseous and soft-tissue techniques for reconstruction of the hip, depending on the pelvic anatomy and deformity, but most on the experience of the treating surgeon [5–11].

The most serious types of dysplasia with severe deformities of the proximal femur following subluxation and dislocation, as well as relevant shortening of the adductor muscles are connected to neuromuscular disorders [12, 13].

In up to a total of 60% of patients with cerebral palsy (CP), NDH was shown by the literature [14]. For that reason, surgical realignment of the pelvic geometry is necessary in hips with a progression of Reimers Migration Index (RMI) to prevent further lateralization and following complications such as dislocation, pain, and impairment [15–17].

Apart from the necessity of open reduction, surgical procedures such as femoral and pelvic osteotomies and lengthening of tendons and muscles may be used to treat hips with NDH [18].

Surgical procedures in ambulating patients are more demanding than the non-walking infant because of progressive contraction of extraarticular soft tissue, femoral anteversion, acetabular dysplasia, and constriction of the hip capsule [8].

In those patients, a combined approach to the reconstruction of the genuine pelvic geometry by usage of osseous and soft-tissue techniques is needed [19, 20].

Despite a lack of data of randomized controlled trials, stepwise or two-stage strategies for reconstruction of the pelvic geometry, starting with open reduction, and varisation–derotation osteotomy (VDRO), followed by osteotomy of the ilium in a second session, are suggested by some surgeons [21].

Due to this lack of mid- to long-term results, it is unclear whether the combined approach or a stepwise strategy is superior in patients with sub- and dislocation of the hip, concerning long-term sustainability of the reconstructed pelvic geometry.

The main aim of this study was to evaluate the mid-term outcome concerning pelvic geometry and complications after pelvic reconstruction in a large cohort of one center for pediatric orthopedics using common osseous and soft-tissue techniques.

The radiologic improvement of the data for pelvic geometry after hip reconstruction treated with osteotomy of the ilium ± femoral osteotomy ± soft-tissue techniques in patients with DDH, NDH, and LCPD was of interest as well as the occurrence of adverse events following surgery.

## Methods

The trial was designed as a retrospective study and approved by local ethics committee. Clinical records of children (age: 2–19 years) with DDH, NDH, and LCPD were screened for pelvic osteotomies (± open reduction, femoral osteotomy, soft-tissue procedures) including from 2008 to 2018 at a University Hospital in central Europe (86 patients). Patients who did not receive a spica cast immobilization postoperatively according to the clinical standard (3 patients) were excluded from further analyses, as well as patients for whom recent (year 2020) radiologic data could not be obtained (lost to follow-up; 10 patients).

Following inclusion and exclusion criteria, 73 children (male/female: 36/37; 84 hips) with a mean age of 8.16 ± 5.83 years at index surgery could be identified. All children were treated by a single pediatric orthopedic surgeon.

The mean timespan from index surgery until follow-up examination was 4.9 years (SD 2.9).

Patients with cerebral palsy were classified according to Gross Motorfunction Classification System (GMFCS). There were 0 type I, 1 type II, 3 type III, 2 type IV, and 16 type V.

Underlying disease and procedures of all cases can be seen in Table 1.

**Table 1:** Hips and procedures

	DDH	NDH	Perthes
*N* (hips)	31	25	28
Age at surgery	5.8 y; 2.9–17; SD 5.7	12.5 y; 5.6–17.8; SD 6.2	7.6 y; 5.2–10.7; SD 1.7
m:f	5:21	11:11	22:3
Right:left	12:9	12:7	9:13
Bilateral	5	3	3
Reimers <25%	0	0	8
Reimers 25–40%	4	0	20
>40%	27	25	0
Tönnis I	0	n.a.	n.a.
II	8	n.a.	n.a.
III	11	n.a.	n.a.
IV	12	n.a.	n.a.
Catterall I	n.a.	n.a.	0
II	n.a.	n.a.	0
III	n.a.	n.a.	12
IV	n.a.	n.a.	16
Herring A	n.a.	n.a.	4
B	n.a.	n.a.	10
C	n.a.	n.a.	14
Surgical procedure in detail			
Femoral osteotomy	31	25	28
Osteotomy of ilium	27	16	12
Salter osteotomy	7	2	12
Chiari osteotomy	2	2	
Pemberton osteotomy	18	12	
Combined osteotomy	27	16	12
Psoas tenotomy		9	1
Adductor tenotomy		8	
Open reduction	16	12	
Hamstring lengthening		7	
Lengthening of extension mechanism		1	

Radiological findings for indicating surgical procedures were Reimers Migration Index (RMI) [22, 23] 40% or higher or 25–40% with progression, Tönnis classification [24] II, or higher or Acetabular Index (AI) above the Tönnis standard.

The operations were carried out under general anesthesia, using fluoroscopy on a radiolucent table. The patient was bedded in supine position with a foam pad under the ilium to allow mild elevation of the side to operate. The whole lower extremity and the ipsilateral side of the pelvis were washed sterile and draped in sterile cloth.

Commonly known surgical techniques were used in all cases: Chiari pelvic osteotomy (4 hips) [9], Salter innominate osteotomy (21 hips) [6] and Pemberton acetabuloplasty (30 hips) [7], and varisation–derotation osteotomy of the femur (84 hips) (Tables 1, 2) [11].

**Table 2:** Groups and procedures

	Sole osteotomy (femur)	Combined osteotomy (ilium and femur)
*N* (patients)	29	44
*N* (hips)	29	55
Bilateral	0	11
Gender m:f	9:20	28:16
Age at surgery	10.7 y; 5.0–17.8 y; SD 6.4	7.8 y; 2.9–15.1 y; SD 5.3
Location	10 right, 19 left	21 right, 12 left, 11 bilateral
Procedures (*n* = hips)		
Femoral osteotomy	29	55
Osteotomy of ilium	0	55
Salter osteotomy	0	21
Chiari osteotomy	0	4
Pemberton osteotomy	0	30
Psoas tenotomy	6	4
Adductor tenotomy	5	3
Open reduction	1	27
Hamstring lengthening	0	7
Lengthening of extension mechanism	0	1

The surgical approach to the ilium and for open reduction was an anterior approach described by Smith-Petersen [25]. The apophysis of the iliac bone was incised and shoven back to expose the Ilium. The direct lateral approach to the proximal femur was used for the varisation–derotation osteotomy.

Evaluation whether open reduction has to be performed in subluxated hips was done using fluoroscopy directly preoperative. Open reduction was performed in cases of DDH (*n *= 16) and NDH (*n *= 12), when an abduction of the hip of 30° did not lead to centering of the hip or complete luxation with RMI > 100% was observed. In all cases of open reduction, a combined femoral and iliac osteotomy was performed.

In general, in cases of LCPD, open reduction was not performed due to the known entity of the disease. Indications for surgery were lateral subluxation, metaphyseal cyst-like changes, lateral calcification, horizontalization of the growth plate, and Gage sign. Preoperative fluoroscopy in abduction of the hip of 30° was performed to show hinge abduction, which was considered to be a contraindication for the common techniques described above. Whenever, following a femoral osteotomy, an additional iliac osteotomy was needed to improve the containment, the technique of Salter was used to pull the ilium in anterolateral direction to cover the femoral head.

In 55 cases, the techniques above described were combined to reconstruct the pelvic geometry. Three K-wires were used to fix Salter and Chiari osteotomies, and Pemberton osteotomies were carried out in press-fit technique.

Osteosynthetic metal used to hold the femoral osteotomy was a standard 90° AO blade plate (66 hips) or a 90° locking cannulated blade plate (18 hips).

In all cases, the cast was applied directly postoperative under general anesthesia by the surgical staff, including one senior surgeon, one junior surgeon, two theater nurses, and two casting professionals. Surgeons and nurses kept the pelvis and lower extremities in the required position, while the casting professionals applied two layers of cotton, followed by at least two layers of plaster. The reconstructed extremity was held in a long leg cast, the contralateral side in a short leg. The hardening cast was split on the operated side.

The position of the extremity operated on was 10° of flexion as well as 10° of inwards rotation of the hip and 20–30° of abduction of the hip.

To keep abduction and for stabilization reasons, the thighs were connected by a wooden rod.

According to the postoperative treatment protocol, immobilization was maintained for 6 weeks, followed by physiotherapeutic measures until full mobilization.

Adverse events and complications recorded during the whole follow-up period were analyzed by screening of the fully available medical documentation. Unscheduled readmissions and contacts in the outpatient clinic were counted. Comparison between DDH, NDH, and LCPD concerning adverse events was performed.

### Radiological analysis

The radiographic hip geometry was measured twice each by author LP to reduce the probability of error and compared statistically.

Radiographic examinations included a standing pelvis AP view and a lateral view pre-, 3 months postoperative, and on follow-up. Hip geometry was measured using AI [26], CE [26], and RMI [26, 27]. CE angle was not measured in patients under the age of 5 years.

Values for distance from the teardrop figure to the center of the femoral head were given for cases of LCPD to show lateralization from 3 months to maximum follow-up. As the teardrop figure appears within 6 months after concentric reduction in patients with DDH and cannot be given reliable in patients with NDH, this values are not given in these cases [28].

### Statistical analysis

Statistical methods were carried out including a profound descriptive epidemiological analysis with arithmetic mean, standard deviation, median at continuous data and scores, and relative frequency for explained variables.

Where necessary, testing for normal distribution (Shapiro–Wilk) was performed to show appropriability of *t* tests. As required, normal distribution was shown for the selected parameters AI, Center Edge Angle (CE angle), and RMI. Radiologically measured variables for pre- and postoperative hip geometry were calculated using a paired samples *t* test (Wilcoxon rank). Sub-group analysis for AI, CE angle, and RMI pre-, 3 months, and on maximum follow-up after surgery was performed, using a paired *t* test (Wilcoxon rank).

**Fig. 1:**
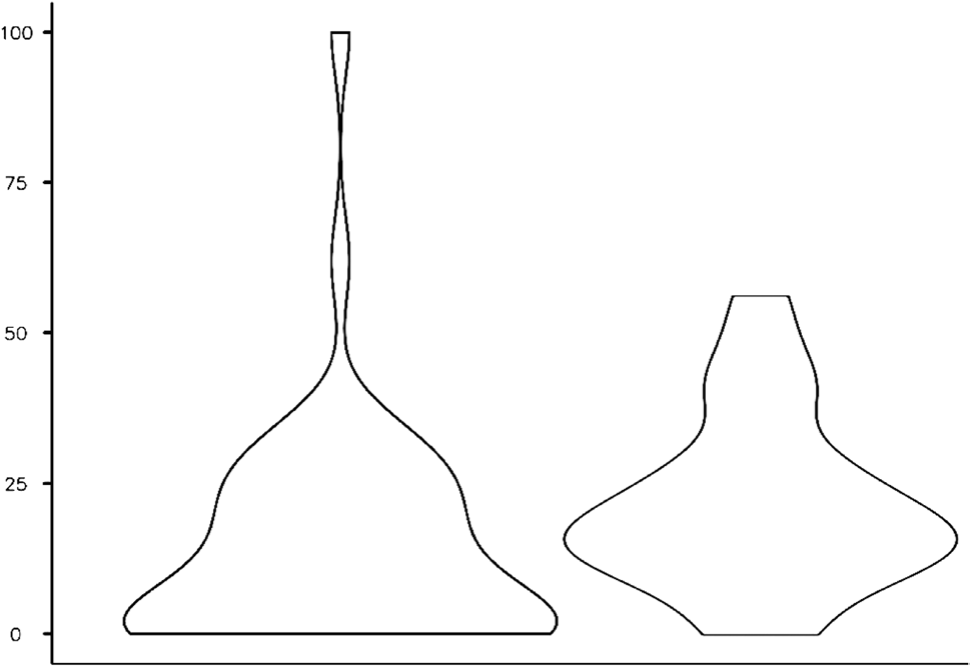
Diagram for residual RMI with combined (left; *n* = 55) and femoral (right; *n* = 29) osteotomy

Statistic independence for variables was calculated using a Chi-square test (Fig. 1).

Values for *P* are given, where necessary, and values of < 0.05 are considered to be statistically significant. Whenever useful, graphics are used to illustrate the statistical results. Microsoft Excel (V 16.43) and Jamovi (V 1.2.27.0) were used for statistical analysis. Calculations were performed on MacOS Big Sur Version number 11.0.1.

## Results

**Figs. 2, 3 and 4:**
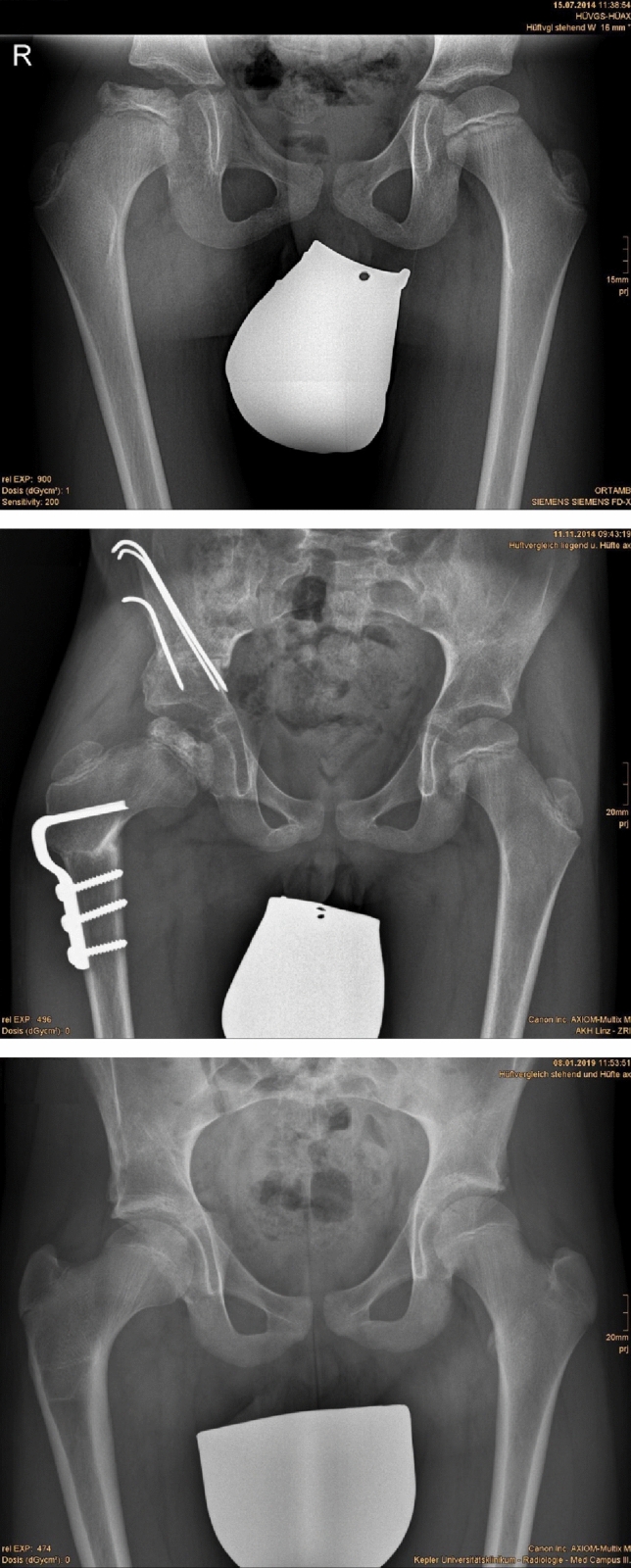
Good result in a 9.5-year-old male after Salter osteotomy and VDRO in LCPD, 3, and 56 months postoperatively

**Figs. 5 and 6:**
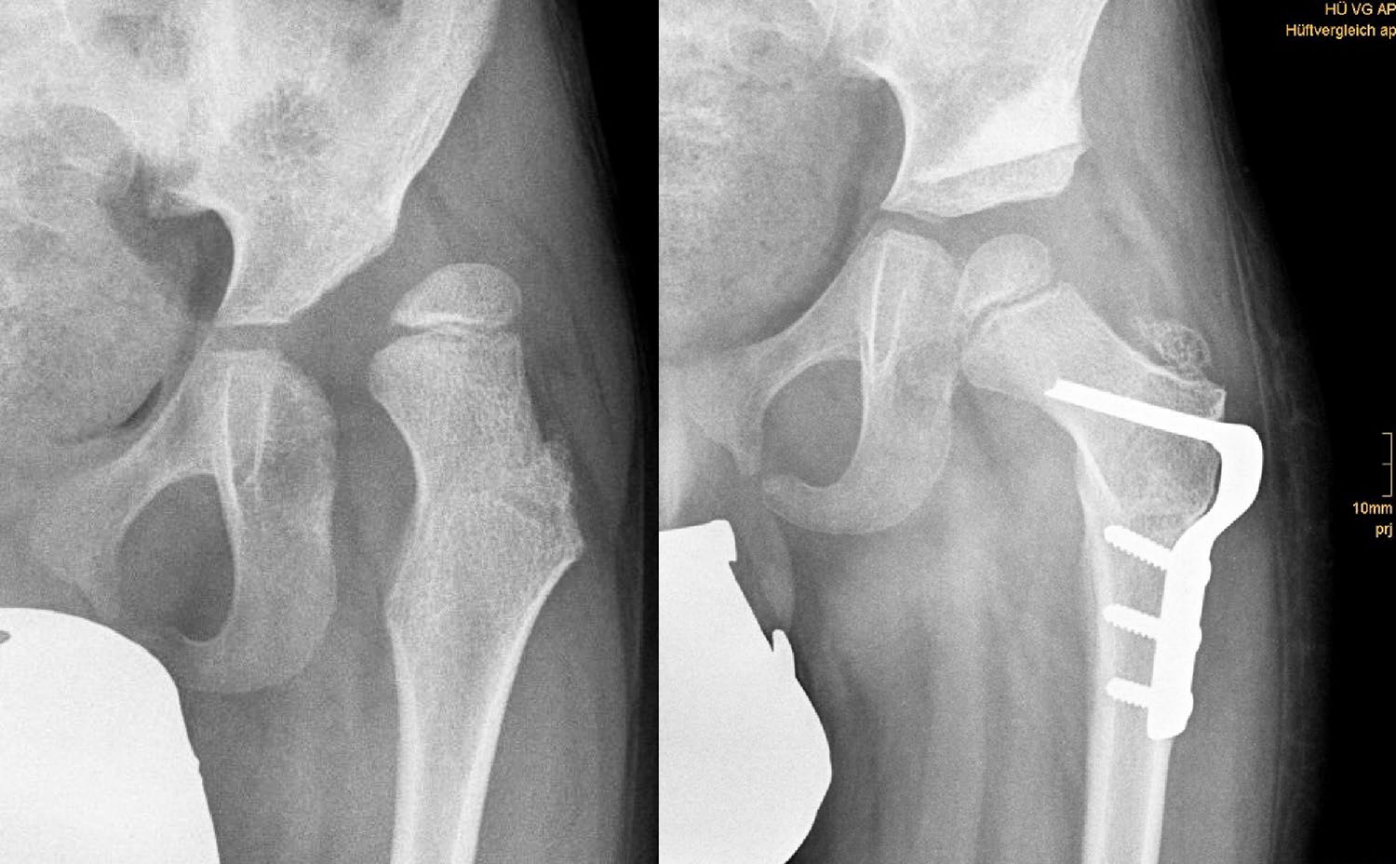
Anterior–posterior X-rays of a 6-year-old boy with neurogenic hip dislocation with excellent results 3 months postoperatively

Pelvic geometry improved significantly in all three groups (DDH, NDH, and LCPD) after hip reconstructive surgery (Figs. 2, 3, 4, 5, 6, Table 3).

**Table 3:** Improvement of hip geometry at maximum follow-up

	AI		*P*-value	CE		*P*-value	RMI		*P*-value
	Pre	Max. f.-up		Pre	Max. f.-up		Pre	Max. f.-up	
DDH (31)	33.9 ± 7.3	19.7 ± 5.3	*P *< 0.001	6.8 ± 16.5	31.6 ± 7.7	*P* < 0.001	80.2 ± 27.9	7.6 ± 10.1	*P* < 0.001
NDH (25)	29.6 ± 6.1	20.5 ± 9.1	*P* < 0.001	11.5 ± 16.8	24.4 ± 12.7	*P* < 0.001	78.0 ± 21.4	22.8 ± 19.8	*P* < 0.001
LCPD (28)	19.0 ± 5.3	19.1 ± 6.5	*P* = 0.101	25.7 ± 6.1	30.1 ± 8.4	*P* < 0.001	27.3 ± 8.3	14.9 ± 11.3	*P* < 0.001
Overall (84)	28.8 ± 9.6	18.3 ± 7.6	*P* < 0.001	11.2 ± 20.1	27.8 ± 15.3	*P* < 0.001	40.6 ± 16.1	15.4 ± 16.0	*P* < 0.001

These results for AI and CE were sustainable until maximum follow-up, but a progression of lateralization of the femoral head (RMI) was noticed.

RMI showed a progressive lateralization in all patient groups at maximum follow-up compared to the results 3 months after surgery (6.1 ± 9.0% vs. 15.4 ± 16%; *P* < 0.001) (Table 4).

**Table 4:** Development of hip geometry from 3 months postop to maximum follow-up

	AI		*P*-value	CE		*P*-value	RMI		*P*-value
	3 months	Max. f.-up		3 months	Max. f.-up		3 months	Max. f.-up	
DDH (31)	19.3 ± 7.3	19.7 ± 5.3	*P *= 0.302	34.3 ± 7.5	31.6 ± 7.7	*P* = 0.822	2.7 ± 6.8	7.6 ± 10.1	*P* = 0.005
NDH (25)	20.8 ± 8.9	20.5 ± 9.1	*P* = 0.617	25.4 ± 11.9	24.4 ± 12.7	*P* = 0.571	13.7 ± 15.3	22.8 ± 19.8	*P* = 0.041
LCPD (28)	16.7 ± 5.8	19.1 ± 6.5	*P* = 0.101	32.9 ± 6.5	30.1 ± 8.4	*P* = 0.134	1.7 ± 4.1	14.9 ± 11.3	*P* < 0.001
Overall (84)	19.1 ± 7.6	18.3 ± 7.6	*P* = 0.171	30.7 ± 9.9	27.8 ± 15.3	*P* = 0.130	6.1 ± 9.0	15.4 ± 16.0	*P* < 0.001

Analysis of the three indications for pelvic reconstruction showed a deterioration of RMI in 9 of 31 hips with DDH, 9 of 25 hips with NDH, and 11 of 28 hips with LCPD. Procedures prone to worsen were combined femoral and iliac osteotomy as well as sole femoral osteotomy, despite further analysis showed a significantly lower RMI in hips with osteotomy of the iliac bone than without at maximum follow-up (13.9 ± 19% vs. 26.9 ± 18.5%; *P* = 0.006).

Sub-group analysis revealed that those results did not depend on whether soft-tissue techniques were carried out or not (6.1±9.4% vs. 16.8±18.4%; *P* = 0.068 and 6.5 ± 12.3% vs. 15.4 ± 15.4%; *P* < 0.001) (Fig. 1).

The pericapsular osteotomy of the ilium (Salter innominate, Pemberton, Chiari) was significantly performed more often in the DDH group than in the NDH group (27 vs. 17; *P* < 0.001).

The mean distance from the teardrop figure to the center of the femoral head showed a progressive lateralization in patients with LCPD from 3 months postoperatively until maximum follow-up (36.5 ± 4.89 vs. 40.6 ± 6.53; *P* = 0.013). Data are corresponding to the values for RMI, which also showed a progression of lateralization (1.7±4.1 vs. 14.9±11.3, *P* < 0.001).

Cases in need of combined osteotomy and open reduction (*n *= 28) compared to those without open reduction (*n *= 16) did not show worse results at maximum follow-up (5.24 ± 9.78 vs. 5.71 ± 8.90; *P* = 0.897).

In cases of LCPD, the outcome according to the classification of Stulberg was 7 type I, 6 type II, 5 type III, 6 type IV, and 4 type V hips [29]. Stulberg types IV and V showed a worse RMI at maximum follow-up compared to types I–III (16.4% vs. 12.8%), but the difference did not reach statistical significance (*P* = 0.458)

Countable complications were seen in 34% of the patients (*n *= 25). Superficial skin lesions were found in 8, deep skin lesions in 3, contraction of adductors in 3, subluxation in 2, dislocations of the cast in 2, osteonecrosis of the femoral head in 2 cases, reluxation in 1, infection of the implanted plate in 1, compliance problem in 1, delayed bone healing in 1, and contraction of knee flexors in 1 case.

Complications occurred more often in the group of NDH (9/22; 40%) than in DDH (7/26; 26.9%), although it did not reach the level of statistical significance (*P* = 0.162).

Unplanned procedures and readmissions needed to treat the adverse events were 5 surgical revisions with 3 repositions, 1 removal of the plate, 3 inpatient treatments for intravenous analgesia and wound care, and 1 admission to the department for high energy shockwave treatment due to delayed healing of the bone.

Four patients were in need for intensive wound care in our outpatient clinic on a regular basis.

## Discussion

Pelvic reconstruction in children and adolescents is a demanding field of surgery for the pediatric orthopedic colleague.

Surgical treatment of ambulating patients is even more difficult caused by shortening of the adjacent soft tissue, progressive dysplasia of the acetabulum, narrowing of the hip capsule, and femoral antetorsion.

Literature reveals several different methods for reconstruction of the hip. The majority of patients are in need for femoral as well as pelvic osteotomies to acquire sufficient congruency of the hip.

To obtain complete roofing of the femoral head to support the rebuilding of the necrosis, the concept of ‘super-containment’ is widely adopted in the treatment of patients with Perthes disease [2–4]. Providing a template in the stage of reossification is necessary to reach congruency and, as a result, to avoid early degeneration of the joint.

Obtaining congruency of the pelvic joint is the crucial aim of hip reconstructive surgery under usage of pericapsular osteotomy in combination with or without varisation–derotating osteotomy and soft-tissue techniques.

As suggested by Huh et al. in 2011, different technical approaches to the cases with dysplastic hips are possible. A sequential approach to surgical management of dislocated hips by first combining VDRO with open reduction and soft-tissue techniques and secondary performing an osteotomy of the ilium if needed was proposed [21].

The type of technique, in the first line, depends on the main pathologies of the pelvic joint, including osseous and soft tissue. Secondary, the concept of reconstruction is strongly connected to the surgeon’s personal experience. As shown in the current literature, a universally valid way how to treat dysplastic hips has not been adopted.

The aim of this current observational study was to evaluate the mid-term radiological outcome of hip reconstructive surgery concerning pelvic geometry in children with DDH, NDH, and LCPD under usage of iliac/femoral osteotomy combined with soft-tissue techniques.

In the cohort presented, the surgical results for hip geometry under usage of acetabuloplasty as described by Pemberton, pericapsular osteotomy of Salter, Chiari osteotomy, and varisation–derotation osteotomy can be compared to the previously published data available in the literature.

84 surgically treated hips in children with developmental and neuromuscular dysplasia of the hip as well as Legg–Calvé–Perthes disease were included in this observational study. For the three groups mentioned, it was possible to present an improvement in hip geometry at a mean follow-up of 4.9 years after hip reconstructive surgery.

The mean preoperative RMI measured 40.6% 15.4% at follow-up. Angles for AI (28.8 and 18.3) and CE (11.2 and 27.8) improved in a comparable way.

The values from the 3-month follow-up compared to the maximum follow-up revealed that RMI showed a progressive lateralization (6.1/15.4, *P* < 0.001), where AI and CE did not change with statistical significance.

Those findings were neither dependent on different patient groups (DDH, NDH, and LCPD) nor on the usage of soft-tissue techniques in combination with osseous procedures. The crucial finding concerning surgical techniques was the statistically significant lower RMI when a combined femoral and iliac osteotomy was performed.

Therefore, data suggest that the procedures involving an osteotomy of the iliac bone are appropriate to establish congruency of the pelvic joint in children and facilitate a sustainable basis for further osseous development of the pediatric hip.

Analysis revealed that patients with NDH have less benefit from surgical procedures concerning RMI compared to patients with DDH (22.8% vs. 7.6%; *P* = 0.011). Data for residual RMI are consistent to previously published literature in children with NDH [20]. More detailed search for correlations between residual RMI and used surgical techniques revealed that combined osteotomy of the femur (VDRO) and the Ilium (Salter innominate, Pemberton, Chiari) leads to a lower the residual RMI (13.9±19% vs. 26.9±18.5%; *P* = 0.006).

**Figs. 7 and 8:**
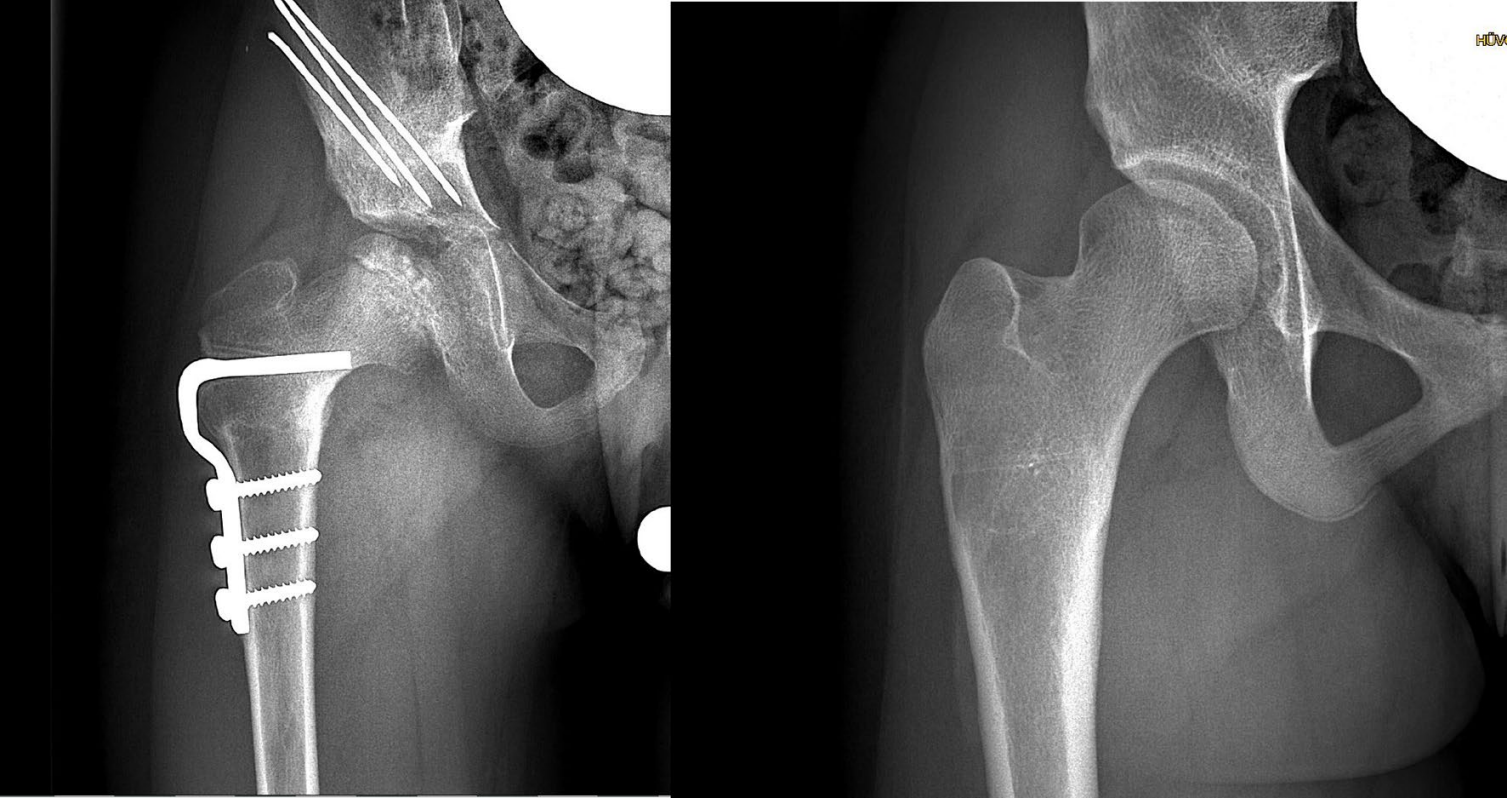
Anterior–posterior X-rays of an 8-year-old girl with LCPD with deterioration of pelvic coverage 7 years postoperatively

Nevertheless, a deterioration of RMI was seen in all three basic entities (DDH, NDH, LCPD), independent from the surgical technique. Reasons for this observation are likely to be multifactorial: dysmorphic femoral head (DDH, NDH, and LCPD), abnormal anteversion of the femoral neck (DDH, NDH), muscular imbalance (NDH), remodeling and enlargement of the femoral head (LCPD), and insufficient anterior coverage (DDH, NDH, and LCPD). Some of these factors may be addressed intraoperatively, such as the femoral anteversion (VDRO), the anterior coverage (Salter osteotomy with anterolateral displacement of the iliac bone), and muscular imbalance (soft-tissue release in NDH). Developments unable to be influenced are the dysmorphic femoral head and the remodeling with enlargement of the femoral head (Figs. 7, 8). Shown by the authors in this study, the mid-term results for centralization of the femoral head may be improved by combined femoral and iliac osteotomy compared to sole femoral osteotomy in alle three groups.

As a main result, the study showed a connection between an osteotomy of the ilium and the sustainable improvement of RMI at maximum follow-up.

In a majority of 27 out of 31 cases with DDH, an osteotomy of the ilium was performed, whereas this number was 17 out of 25 in NDH. It has to be assumed that this contrast leads to the difference in postoperative RMI within these two groups.

As a consequence, the authors aim to perform osteotomies of the iliac bone more likely in future surgical procedures to achieve congruency of the pelvic joint.

With a follow-up period of 38 months, Zhao et al. showed in 2012 better outcomes in DDH for the combined femoral and ilium osteotomy compared to the simple ilium osteotomy. Parameters of interest were redislocation rate, avascular necrosis of the femoral head, and joint stiffness. Data for RMI, as in this study, were not given [30].

In 2009, Al-Ghadir et al. presented a study in patients with DDH, which revealed a lower rate for revision surgery in a group with combined femoral and ilium osteotomy in contrast to the VDRO alone [31].

Additional favorable results for the combined femoral and iliac osteotomy concerning hip geometry in DDH were presented by several authors such as Czubak et al., El-Sayed et al., and Al-Ghamdi et al. [8, 32, 33].

For patients with NDH, Braatz el al. published data for 91 complex hip reconstructions. The preoperative RMI of 100% improved to 5.6% directly postoperative and 14.0% after 7.7 years [20]. An average increase of RMI from 5.6% to 14% was observed within the follow-up period; nevertheless, a combination of surgical procedures was successful in severe cases of NDH.

Summarizing results for patient groups with LCPD treated with Salter osteotomy, Park et al. (CE angle 19.7 vs. 29.6°), as well as Ishida et al. (CE angle 17.9 vs. 35.2°) were able to show reliable results concerning hip joint geometry and re-operation rate [34, 35]. RMI was not reported in those publications.

Apart from sustainable mid- to long-term results concerning pelvic geometry, a complication rate of 34% was noticed. The majority of the complications were easy to treat, which is comparable to the known literature, but much too high to ignore [36, 37]. Complications were observed more often in patients with NDH, although the difference did not reach statistical significance. Cast-associated complications are a matter of ongoing discussion about the proper way of immobilization and result in a broad variety of cast applications or alternative techniques [37, 38].

Limitations of our study are the mid-term follow-up period from surgery to the measured a-p X-ray.

Especially in NDH, annual follow-ups are required to reevaluate the situation of the hip as literature shows a re-operation rate of up to 40% [39].

Further limitations of our study are the little number of 73 included patients and the heterogeneity of the group, as the procedures described are not high-volume surgery, but necessary in a small number of demanding cases.

As to the knowledge of the study group, this is the first trial communicating superiority of the combined osteotomy concerning residual RMI at a follow-up of 4.9 years postoperatively.

Longer follow-up periods are in need to prove the long-term effects and will be communicated by the study group.

## Conclusion

This study was able to summarize that the reconstructive methods used to improve pelvic geometry in patients with DDH, NDH, as well as in LCPD. A one-step combined femoral and iliac osteotomy leads to favorable results than a sole femoral osteotomy at short- to mid-term follow-up. As already shown for patients with NDH, data for RMI are not as steady as for AI and CE, also in patients with DDH and LCPD.

## Data Availability

The datasets used and/or analyzed during the current study are available from the corresponding author on reasonable request. Only authors have full access to the dataset.

## Abbreviations

DDH: Developmental dysplasia of the hip
NDH: Neurogenic dislocation of the hip
LCPD: Legg–Calvé–Perthes disease
VDRO: Varisation–derotation osteotomy
RMI: Reimers migration index
AI: Acetabular index
CE: Center edge angle
